# Correlation Between BMI and Kidney Tumor Lateralization: Insights into Survival and Risk Factors

**DOI:** 10.3390/cancers16244139

**Published:** 2024-12-12

**Authors:** Mateusz Czajkowski, Michał Falis, Anton Żawrocki, Magdalena Sternau, Andrzej Lubiewski, Magdalena Rytlewska, Marcin Matuszewski

**Affiliations:** 1 Department of Urology, Medical University of Gdańsk, 80-214 Gdańsk, Poland; msternau@gumed.edu.pl (M.S.); marcin.matuszewski@gumed.edu.pl (M.M.); 2 Faculty of Medicine, Medical University of Gdańsk, 80-214 Gdańsk, Poland; m.falis@gumed.edu.pl; 3 Department of Pathology, Specialist Hospital in Wejherowo, 84-200 Wejherowo, Poland; anton.zawrocki@gumed.edu.pl; 4 Department of Emergency Medicine, Faculty of Health Sciences, Medical University of Gdańsk, 80-214 Gdańsk, Poland; andrzej.lubiewski@gumed.edu.pl; 5 Department of Rheumatology, Clinical Immunology, Geriatrics and Internal Medicine, Medical University of Gdańsk, 80-214 Gdańsk, Poland; magda.rytlewska@gumed.edu.pl

**Keywords:** kidney cancer, lateralization, obesity, overweight, hypertension

## Abstract

**Background**: Kidney cancer accounts for approximately 2% of all diagnosed cancers and fatalities worldwide, and a notable increase in its incidence has been observed in recent years. Previous studies have identified various risk factors for renal cell carcinoma (RCC), including age, gender, smoking, hypertension, overweight, and obesity. However, limited information is available regarding the correlation between RCC risk factors and tumor lateralization. **Objectives**: To investigate the relationship between body mass index (BMI) and the lateralization of kidney tumors in patients undergoing surgery for renal cell carcinoma. Moreover, we aim to evaluate the impact of lateralization of malignant kidney tumors on overall survival (OS) and cancer-specific survival (CSS). **Materials and Methods**: This single-center study included 287 patients who underwent surgical treatment for kidney tumors between January 2016 and December 2019. The patients were allocated into the following groups based on their BMI: normal (18.5–24.99 kg/m^2^), overweight (25–29.99 kg/m^2^), or obese (≥30 kg/m^2^). The study collected demographic and histopathological data, as well as patient history, including risk factors such as smoking and hypertension. **Results**: Right-sided kidney tumors occurred more frequently (55.05%, n = 158) than left-sided ones (44.95%, n = 129). A statistically significant relationship (*p* = 0.04731) was observed between BMI and the frequency of right-sided kidney tumors in the overweight group (70 vs. 43 cases). There was no correlation between BMI and the occurrence of kidney tumors in the normal BMI and obesity groups. Furthermore, the analysis revealed no association between the lateralization of kidney tumors, smoking, and hypertension. The 5-year survival rate was 62%, with a mean follow-up duration of 104 months (approximately 8.5 years). No statistically significant difference was observed between the right- and left-sided cancer groups, with survival rates of 58% and 66%, respectively (*p* = 0.652). **Conclusions**: This study highlighted that right-sided kidney tumors occurred significantly more frequently in overweight individuals in our cohort of patients. No association was observed between lateralization of kidney cancer and overall survival (OS) or cancer-specific survival (CSS).

## Simple Summary

In light of the steady increase in kidney cancer cases and the rapid growth of the number of people with excessive body mass, extending the knowledge about their correlation emerged as the main goal of our study. We focused on the lateralization of tumors among individuals with varying body mass indices. Additionally, this investigation examined other well-established risk factors, such as hypertension and smoking, and their influence on tumor lateralization. Furthermore, we assessed the effect of renal cell cancer lateralization on patient survival. The results of our analysis may potentially contribute to the development of prevention programs for specific groups of risk factors, earlier detection of kidney cancers, and improvement of prognosis based on tumor location as one of the criteria.

## 1. Introduction

Kidney cancer accounts for approximately 2% of all diagnosed cancers and deaths worldwide [1]. Its prevalence has been increasing in recent years, with approximately 400,000 cases diagnosed per annum [2]. Renal cell carcinoma (RCC) occurs twice as frequently in men as in women [3,4]. Although RCC typically affects individuals between the ages of 60 and 70, there is an alarmingly increasing number of RCC cases among young people, particularly in Europe [5]. Previous studies have highlighted several confirmed risk factors, including cigarette smoking, hypertension, being overweight, and obesity [6,7]. Early-stage diagnosis of kidney cancer provides more treatment options, including less burdensome methods for the patient, such as nephron-sparing surgery, which allows for preservation of kidney function [8,9]. Early diagnosis also notably increases the 5-year survival rate (93% for early-stage cancer and 12% for metastatic cancer) [10]. Additionally, according to Scoll et al. [11], a tumor size <4 cm correlates with better relative patient survival than larger tumors (>7 cm). Previous studies have suggested that RCC lateralization significantly affects patient prognosis. Specifically, right-sided RCC exhibits less frequent metastasis to lymph nodes and other organs, as well as a lower overall rate of cancer progression than left-sided RCC [12]. With the declining prevalence of tobacco smoking [13], overweight and obesity have become increasingly important factors in the pathogenesis of renal cell carcinoma. This trend is particularly evident in the United States, where the population occurrence of excess body weight increased from 18% to 29% and smoking decreased from 12% to 9% [14]. This increasing number of individuals with overweight and obesity [15] is accompanied by an increase in individuals suffering from hypertension [16]. Considering these global patterns, our study aimed to investigate the relationship between body mass index (BMI) and lateralization of kidney tumors in patients undergoing surgery for renal cell carcinoma. Moreover, a 5-year follow-up study was conducted to evaluate overall survival (OS) and cancer-specific survival (CSS) in relation to the lateralization of the malignant kidney tumors.

## 2. Materials and Methods

This study was conducted from January 2016 to December 2019 at a single tertiary medical center and included 287 patients with kidney tumors of both sexes who underwent surgical treatment. The study was approved by an independent ethics committee (Decision No. NKBBN/370/2016). Patients qualified for a specific surgical procedure based on multiple criteria, including the results of a computed tomography (CT) scan performed before surgery. Patients presenting various additional risk factors were classified based on the primary criterion—body mass index (BMI)—into the following groups: normal BMI (18.5–24.99 kg/m^2^), overweight (25–29.99 kg/m^2^), or obesity (≥30 kg/m^2^). For each patient, we collected demographic and histopathological data and conducted a detailed survey of patient history to identify the occurrence of commonly recognized risk factors (such as smoking and/or hypertension). The Kaplan–Meier method and the Kaplan–Meier method with log-rank test were employed to conduct survival analysis of overall survival (OS) and cancer-specific survival (CSS) in patients with malignant kidney tumors.

## 3. Results

The analysis presented below was based on data from 287 patients who underwent surgery for kidney tumors at a single tertiary medical center. The experimental group had a median age of 64 years (range, 28–88 years), a median height of 172 cm (142–200 cm), a median weight of 80 kg (30–144 kg), and a median BMI of 27 kg/m^2^ (17–48 kg/m^2^). In this cohort, 89 individuals (31.01%) had normal BMIs, 113 (39.37%) were overweight, and 85 (29.61%) were obese. Further information on the cohort characteristics is provided in Table 1.

**Figure 1:**
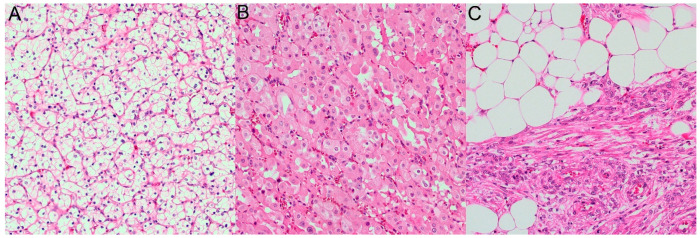
Typical histopathological images of: (**A**) clear-cell renal cell carcinoma, (**B**) oncocytoma, and (**C**) angiomyolipoma in hematoxylin and eosin staining, ×200.

Most patients (55.05%, n = 158) had a kidney tumor located on the right side, whereas in the remaining individuals (44.95%, n = 129), the tumor was on the left side. Histopathological examination revealed 257 cases of clear-cell renal cell carcinoma (89.55%), 18 of oncocytoma (6.27%), and 12 of angiomyolipoma (4.18%). Typical histopathological images of each type are shown in Figure 1.

The associations between established risk factors for kidney cancer (smoking, hypertension, and weight disorders) were investigated using data collected through patient surveys.

Most patients were diagnosed with arterial hypertension (65.85%, n = 189) and were non-smokers (64.81%, n = 186). Amongst the smokers (35.19%, n = 101), the median pack-years were 25 (range 2.5–70). Notably, some patients had multiple risk factors in history surveys. In 73 patients, both hypertension and smoking were present (*p* = 0.343), while in 159 individuals, both hypertension and being overweight were observed (*p* = 0.00008). Additionally, in 78 subjects, both smoking and overweight were identified (*p* = 0.66841).

A statistically significant correlation (*p* = 0.005) was identified between the occurrence of hypertension and the emergence of malignant subtypes of kidney cancer. Specifically, clear-cell RCC was more common than the other subtypes (*p* = 0.003). However, no correlation was observed between hypertension and tumor lateralization (*p* = 0.233). Additionally, there was no correlation between smoking and malignancy, histopathological subtype, or tumor lateralization. Furthermore, no correlation was observed between overweight and histopathological subtypes or the malignancy of kidney tumors. Further details regarding the relationships between the risk factors and tumor lateralization are shown in Table 2.

**Figure 2:**
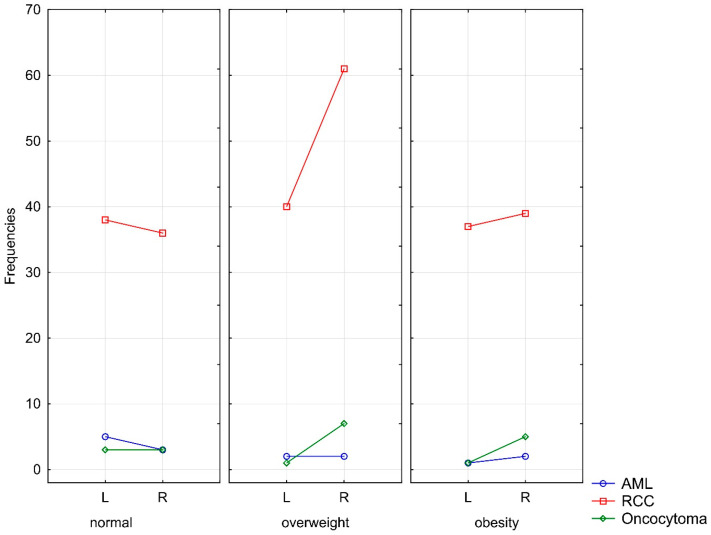
Relationship between different histopathological subtypes of kidney tumors and lateralization in patients with normal BMI, overweight, and obesity. L—left-sided; R—right-sided.

The analysis identified a significant relationship (*p* = 0.047) between body mass index (BMI) and the incidence of right-sided kidney tumor in the overweight group (BMI 25–29.99 kg/m^2^; 70 vs. 43 cases). However, a similar trend was not observed in the normal and obese groups (Figure 2).

Various factors were considered when deciding on the optimal surgical procedure, including the results of CT scans performed prior to surgery. Nephron-sparing surgery (NSS) was the selected treatment in 140 patients (48.78%), followed by nephrectomy in 137 patients (47.74%) and other therapeutic approaches in the remaining 10 patients (3.48%). No statistically significant correlation was detected between the occurrence of hypertension, overweight, or smoking and the type of kidney tumor treatment employed.

**Figure 3:**
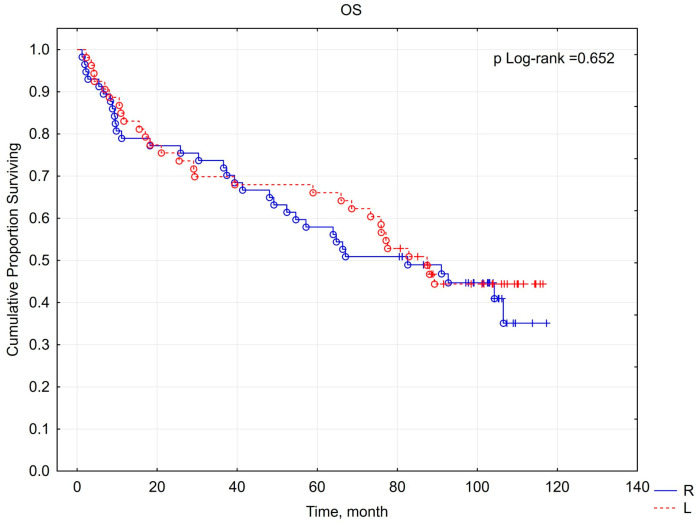
Relationship between lateralization of renal cancer and overall survival (OS).

**Figure 4:**
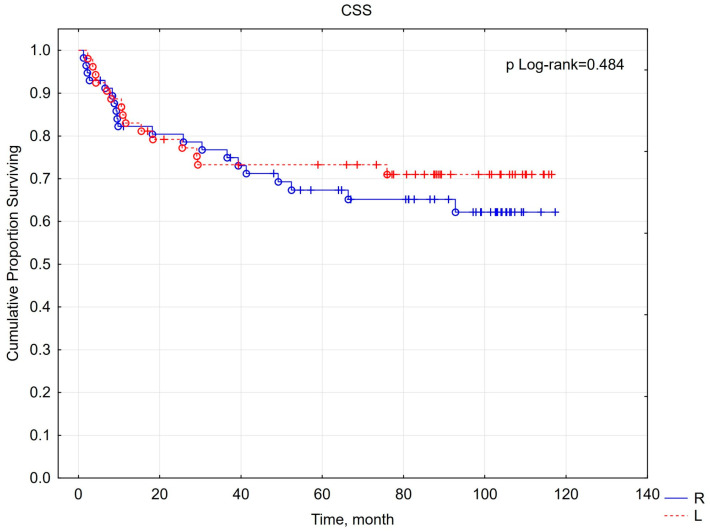
The relationship between lateralization and cancer-specific survival (CSS).

**Figure 5:**
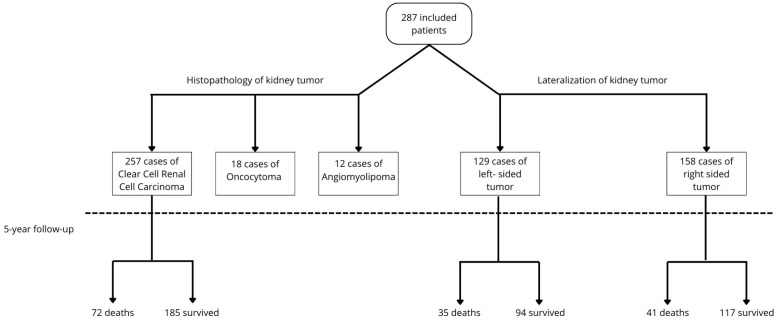
Flow chart of the study group with 5-year follow-up results (Chart created with Canva).

Following a minimum 5-year follow-up period, an assessment of overall survival and cancer-specific survival was conducted among patients with malignant tumors. The 5-year survival rate was 62%, with a mean follow-up duration of 104 months (approximately 8.5 years). No statistically significant difference was observed between the right- and left-sided cancer groups, with survival rates of 58% and 66%, respectively (*p* = 0.652). Similarly, CSS rates were not significantly different between the right-sided (67%) and left-sided (73%) groups (*p* = 0.484) (Figure 3 and Figure 4). In the study cohort, renal cancer was identified as the primary direct cause of mortality (42 cases, 57.53%). Additional causes of death included sepsis (five cases, 6.85%); lung cancer (four cases, 5.48%); COVID-19 infection (four cases, 5.48%); heart failure (four cases, 5.48%); stroke (three cases, 4.1%); sudden cardiac arrest of unknown etiology (two cases, 2.73%); and single cases (1.37% each) of Alzheimer’s disease, complications from type II diabetes, stomach cancer, kidney failure, bladder cancer, liver cirrhosis, adult-type nephroblastoma, and hemorrhagic shock. The flow chart of the study group with the 5-year follow-up results is shown in Figure 5.

## 4. Discussion

Among the well-established yet modifiable risk factors for kidney cancer, hypertension, tobacco smoking, and obesity are noteworthy [17,18]. Obesity and overweight represent some of the most pressing global challenges. Studies indicate that up to one-third of the world’s population is classified as either overweight or obese [19], and the number is increasing. We observed a similar trend in our study group, in which the number of individuals with excessive body weight significantly exceeded the number of smokers (218 vs. 102, respectively). In our study, we used BMI as an objective measure because of its simplicity and usefulness in categorizing patients, and its overall correlation with the frequency of neoplastic changes has been confirmed in previous studies [20,21]. BMI has been found to be positively associated with an increased risk of kidney cancer. According to Scelo et al. [18], for each additional 1 kg/m^2^ in BMI, the risk increased by 4%, and for every 5 kg/m^2^, it increased by as much as 25%. Furthermore, the authors demonstrated that excessive body weight during adolescence significantly increased the likelihood of kidney cancer later in life, even in cases of weight loss later in life. It is estimated that up to 26% of kidney tumors are correlated with an elevated BMI [22]. However, during statistical analysis, we did not identify a correlation between arterial hypertension or smoking and the lateralization of kidney tumors. The localization and degree of tumor advancement are expected to be multifactorial and interconnected. Nevertheless, a noteworthy finding in our investigation was the absence of a correlation between obesity (BMI ≥ 30) and lateralization of kidney tumors.

Kidney cancer often exhibits an initially asymptomatic course before reaching an advanced stage [23]. The appearance of symptoms typically correlates with a more aggressive tumor histology and a higher disease stage [24,25]. Despite the increasing availability of imaging studies that aid in the early detection of cancers, approximately 17% of patients develop distant metastases at the time of diagnosis [26]. Ultrasonography has emerged as a cost-effective and widely accessible method for early-stage kidney cancer detection [27,28]. Therefore, the feasibility of introducing it as a screening method, especially in individuals with an elevated BMI, and consequently, an increased risk of kidney cancer, should be considered. However, one should be aware of the limitations of this method in diagnosing small tumors (<3 cm) due to the high number of false-positive results [29]. In such cases, further evaluation should rely on other imaging techniques, including CT scans, for which it is necessary to expose the patient to X-ray radiation, which incurs additional costs for the healthcare system. Further research on the use of ultrasonography, including contrast-enhanced techniques, may improve the assessment of changes in the kidneys [30,31]. The discovery of currently unknown early-stage kidney cancer markers would provide an important diagnostic tool, although further research is needed to achieve this important goal.

The primary focus of our study was to investigate lateralization in kidney cancer. Furthermore, with respect to malignant renal neoplasms, we sought to examine the association between tumor lateralization and its influence on overall survival (OS) and cancer-specific survival (CSS). Studies have revealed lower TNM staging, smaller tumor diameters, and greater median survival rates in patients with right-sided kidney cancer [32]. Additionally, there is a higher percentage of small tumors on the right side, allowing treatment choices that affect patients to a lesser extent, such as nephron-sparing surgery [33]. Our results demonstrated a statistically significant association between being overweight and the occurrence of right-sided kidney tumors. Early detection through screening in individuals with elevated BMI, coupled with determination of the tumor’s right-sided localization, may contribute to improved prognosis assessment and treatment choice, including less invasive methods.

Unfortunately, the literature on kidney tumor lateralization and its impact on survival and prognosis is limited and conflicting. Contrary results were presented by Roychoudhuri et al. [34], who found no statistically significant differences in the frequency of occurrence and survival of RCC based on lateralization. Additionally, Russo et al. [35] did not find a statistically significant difference in the 5-year progression-free survival and overall postoperative survival between left- and right-sided RCC. The findings of both investigations align with our results, wherein a significant association was not observed between the lateralization of malignant renal neoplasms and overall survival (OS) or cancer-specific survival (CSS).

The issues of tumor lateralization and stage may potentially depend on multiple factors, which could explain the discrepancy between the results of different studies. There are differences between the right and left kidneys in both vascular anatomy and position. The left renal vein has greater collateral circulation connecting the lumbar, gonadal, and adrenal veins, which may serve as a pathway for metastases and explain why left-sided RCC commonly metastasizes to the lungs [12]. Differences in lymphatic drainage from both the kidneys may also influence the timing of metastasis [32]. Additionally, the dorsal position of the left kidney may pose greater diagnostic challenges during ultrasonography and contribute to later detection of changes [32].

Roychoudhuri et al. [34] conducted an analysis of the literature pertaining to tumor lateralization in paired organs and observed a correlation between organ mass disparities, such as those in the testes and lungs, and the frequency of cancer occurrence. However, they did not demonstrate a similar correlation for kidney cancer despite the inclusion of this organ in the study. Nevertheless, it is worth considering whether this could be one of the factors influencing tumor lateralization in individuals with increased body mass, especially because Grandmaison et al. [36] showed a positive correlation between BMI and kidney mass. Other factors (such as height, gender, and age on organ mass) [37], as well as the possible slight natural asymmetry in size between the left and right kidneys observed during autopsies in both genders [38,39], could influence cancer lateralization. Demonstrating a potential correlation between kidney mass and the frequency of tumor occurrence, considering the tendency for increased mass of this organ in individuals with elevated BMI, could help in developing special diagnostic procedures aimed at detecting neoplastic changes as early as possible.

It is worth noting that the risk factors included in our study are interrelated, as evidenced by the correlation between elevated BMI and higher blood pressure values [40,41]. Our analysis revealed a statistically significant correlation between arterial hypertension and the occurrence of malignant kidney tumors, particularly clear-cell RCC. This finding is particularly important in light of the increasing prevalence of hypertension [16], which doubled, increasing from 650 million to 1.3 billion, between 1990 and 2019. Physiologically, one of the elements regulating blood pressure is the renin–angiotensin system (RAS), particularly the product of this axis, angiotensin II. This molecule contributes to the tumor microenvironment and promotes migration, invasion, proliferation, and angiogenesis [42]. Up to 90% of individuals with malignant hypertension and renal hypertension have elevated levels of angiotensin II [43]. However, studies indicate a better prognosis for these patients compared to those without arterial hypertension, which is associated with a lower T stage, smaller tumor size, and less frequent metastases [44]. This phenomenon could potentially be explained by the more frequent monitoring of such patients, which aids in the earlier detection of changes. Another hypothesis is the use of ACE inhibitors, which lower the angiotensin II levels. Previous studies have revealed that postoperative use of RCC significantly prolongs overall survival, including progression-free and disease-specific survival [45,46]. As such, further research on arterial hypertension treatment, with a particular emphasis on the use of ACE inhibitors, may contribute to reducing the risk of developing kidney cancer and/or improving the chances of already-diagnosed patients.

Our study demonstrated a statistically robust relationship between overweight and right-sided kidney tumors. However, it is worth noting that this correlation was only applied to malignant tumors and was not observed in benign tumors. Therefore, evaluating a patient’s BMI represents an important addition to the initial stages of cancer diagnosis and treatment. Incorporating routine BMI assessments can enhance patients’ overall cancer awareness and vigilance for those with weight disorders. One should also provide general advice for maintaining a healthy weight and educate individuals about adopting a healthy lifestyle, including reducing other risk factors of kidney cancer. Additionally, during the performance of ultrasound examinations in individuals with elevated BMI, healthcare providers should exercise extreme caution and pay particular attention to any lesions that may be present on the right side. Any detected lesions underwent comprehensive examination to unequivocally ascertain their nature.

The main limitation of our study is its design—a single-center, retrospective cohort with a limited number of participants. The findings presented here are derived from a single institution, which may not fully reflect the situation in other settings or healthcare systems offering similar procedures. Additionally, the relatively small study population and retrospective nature of the study may have impacted the reliability of our results. Among other limitations, it is important to highlight the significant heterogeneity within the study group. Patients exhibited various risk factors simultaneously, including tobacco smoking, hypertension, and advanced age. Moreover, we lacked data on the duration of weight disturbances in individual patients, which could have affected the development of other independent risk factors. We recommend that future research on this topic consider conducting prospective, multicenter studies with larger cohorts. A potential avenue for further investigation could involve examining the relationship between body mass parameters and the occurrence of kidney cancer, utilizing ultrasonography as a potential screening tool. Furthermore, additional studies are needed to explore the generalizability of these findings across diverse populations.

## 5. Conclusions

This study highlights that right-sided kidney tumors occurred significantly more frequently in overweight individuals in our cohort of patients. Therefore, the introduction of ultrasonography as a screening method for individuals with elevated BMI has been suggested. No correlation was observed between lateralization and overall survival (OS) or cancer-specific survival (CSS) in malignant tumors.

## Figures and Tables

**Table 1:** Demographic and histopathological data.

Risk Factor	Left-Sided Tumor (n)	Right-Sided Tumor (n)	*p*-Value
Smoking (+)	45	57	*p* = 0.497
Smoking (−)	83	101	
Hypertension (+)	82	106	*p* = 0.233
Hypertension (−)	47	51	
Overweight (+)	90	128	*p* = 0.047
Overweight (−)	38	30	

**Table 2:** Lateralization of kidney tumors according to known risk factors.

Patients	Valid N	Minimum	Maximum	Median
Age [yo]	287	28	88	64
Height	287	142	200	172
Weight	287	30	144	80
BMI	287	17	48	27
**Patients**	**Criteria**	**Count**	**Percent** **[%]**	
Sex	Female	102	35.54	
	Male	185	64.46	
Histological type	AML	12	4.18	
	RCC	257	89.55	
	Oncocytoma	18	6.27	
Nicotinism	Yes	101	35.19	
	No	186	64.81	
Hypertension	Yes	189	65.85	
	No	98	34.15	

## Data Availability

Data are contained within the article.
